# Deals of a feather… Modelling latent classes in R&D collaboration data using finite mixture analysis

**DOI:** 10.1371/journal.pone.0307116

**Published:** 2024-09-06

**Authors:** Troy Neilson, Joshua Byrnes, Nicholas Rohde

**Affiliations:** 1 Centre for Applied Health Economics, Griffith University, Nathan, Queensland, Australia; 2 School of Accounting, Finance & Economics, Griffith University, Nathan, Queensland, Australia; Ozyegin University: Ozyegin Universitesi, TÜRKIYE

## Abstract

This work explores if behaviour-based asymmetries are likely to impact deal valuation in the life sciences by examining positive public sentiment as a proxy for market behaviour when negotiating under asymmetric conditions to examine heterogeneity in research & development collaboration (RDC) deal data. We use public sentiment as a proxy for behaviour along with stage of development-based RDC deal data to search for latent classes in the deal data using finite mixture modelling. The analysis reveals a nuanced picture: public sentiment emerges as a significant predictor of deal value, but only for approximately 15% of the data set. This subset exclusively includes firms in the Preclinical stage, where projects have moved past discovery but are yet to commence human studies. Interestingly, the research finds that sentiment’s impact on deal valuation is particularly pronounced in this stage, suggesting heightened market sensitivity. With recent research demonstrating that knowledge asymmetry and behaviour impact valuation volatility, we take this further by capturing latent classes within the data which demonstrates how behaviour is most influential in deal pricing considerations. We argue that our research demonstrates the impact of asymmetry and market behaviour on a subset of RDCs where products are known, but likelihood of success is difficult to determine.

## 1 Introduction

Policymakers and pharmaceutical partnering executives the world over are approaching the allocation of capital towards biotechnology research and development (**R&D**) with renewed vigour. The financial impacts of the COVID-19 pandemic continue to demonstrate the importance of pandemic preparedness in an ever-increasingly globalised economy [1]. Capital allocation in the context of biotechnology product development usually happens through the mechanism of an R&D collaboration (**RDC**) [2]. This mechanism allows for a stage-wise approach to product development, where a product is generally provided with enough funding to reach the next clinical milestone [3]. At this point, the product is tested in a clinical setting, where a go/no-go decision on further development, and ultimately, further investment, is made. The gap in research can be found where valuation in this paradigm is challenging due to the inherent knowledge asymmetries found in these types of transactions, long drug development timelines (Drug development timelines vary drastically by therapeutic area, the allocation of capital and the urgency to bring the particular product to market. Whilst typical products can take ∼15 years to make it through the regulatory and development frameworks, the COVID-19 pandemic has demonstrated how we can speed development where the need is great…) and the inherently complex nature of scientific product development where human safety is of paramount concern [4–6].

### 1.1 Knowledge asymmetries and decision making

Knowledge asymmetries in this context are underpinned by the fact that trust, and ultimately understanding, between collaborating firms is not symmetrical. Where firms choose, or fail, to disclose for any myriad of reasons, commercial or otherwise, knowledge asymmetries will exist [7, 8]. Bias in these settings tends toward the survivor, with disclosures generally only coming from successful transactions [9]. Knowledge asymmetries are also a natural part of the drug development process, where early-stage products lack the clinical and safety data required to inform the assumptions around safety and efficacy. In this context, disclosures increase as a product progresses through development, and thus, practitioners are better informed to make decisions around valuation [10].

Understanding the theory of bargaining under conditions of asymmetric information when considering RDCs offers a nuanced understanding of the strategic interplay between entities engaged in such partnerships. It illuminates the intricate dynamics that ensue when information is unevenly distributed among stakeholders, affecting decision-making processes and valuations in significant ways. A comprehensive exploration of the completeness of information available to parties involved in RDCs, including firms and investors, would reveal how disparities in knowledge can influence behaviour and valuation judgments. Asymmetric information shapes the bargaining landscape, this framework enriches our comprehension of the valuation mechanisms at play within RDCs. It underscores the critical role that information plays in the negotiation and decision-making processes, providing valuable insights into the complexities of valuing collaborative ventures in sectors characterised by high uncertainty and innovation [4, 8, 11].

Based upon the assumption that complex decisions such as those found in RDCs are heterogeneous in nature, we assume decision makers act with autonomy, seeking to guide decision-making behaviour through the examination of market sentiment as discussed in research by Lecouteux et al., 2015 and Mardjo et al., 2022 [12, 13]. With these assumptions in hand we examine if behaviour-based asymmetries and bias impact RDC valuation through the application of Finite Mixture Modelling (**FMM**) on RDC deal data. We use public sentiment analysis based upon micro-blogging (Twitter) as a proxy for market behaviour. We also examine if the knowledge asymmetries outlined are meaningful in RDC valuations. In section 2 we outline the models traditionally used to value RDC transactions. In section 3 we go on to describe the benefits of using a proxy for market behaviour when modelling RDC values. In sections 4 & 5 we propose our hypothesis and describe the data used for this research. Finally, we follow this with our methodology, results, and then we discuss our findings, their implications, limitations and how this research can be expanded through future inquiry.

## 2 R&D collaboration valuation

Valuation methodologies for biotechnology and pharmaceutical transactions abound and the assumptions of efficient market theory generally hold true where disclosures are full and transparent. In these cases, valuations can rely on information available to the market to inform the valuation process [14, 15]. Where disclosures are not forthcoming, we see that asymmetries and bias born from RDC deal survival exist. Methodologies have been developed to capture these biases and asymmetries and provide the tools needed to value biotechnology transactions where these limitations exist. Yet, whilst the ontologies are plentiful, the preponderance of valuation methodologies can be traced back to two core methods: binomial lattice (decision tree) or risk-adjusted net present value (**rNPV**). Where these methods fail to yield appropriate results, practitioners look to comparable deals to understand the market conditions for deal-doing [3, 16–20].

An rNPV is described in Eq 1, where the methodology accounts for all the future revenues, risks, and costs associated with a biotechnology RDC.

rNPV=PNPVR0-(∑t=0nCiR0Ri) (1)

*P* represents the project’s payoff, *R*_0_ captures the current risk or likelihood of success. *R*_*i*_ represents the risk-adjusted at time *i* and $\frac{C_{i}R_{0}}{R_{i}}$ captures the project’s risk-adjusted costs associated with the project. An *rNPV* is equivalent to an *RDC*s time cost of money over the project’s life cycle, which is otherwise analogue to an IRR, and project-specific risk at that point in the project (*R*_0_). Compounding risks throughout the project culminate early in the project to effectively devalue the project per stage of development based on the ultimate value of the product at the conclusion of the RDC [19].

A binomial lattice based upon the Kellogg and Charnes approach is outlined in Eq 2. Whilst this methodology ultimately breaks down into a series of NPV calculations at a given go/no-go point, it allows the user to map the product life cycle by applying appropriate risk weightings at each stage of development for a detailed schematic of the decision matrix across development, where *i* = 1, …, 5 is a representation of the clinical stages of development: discovery, preclinical, phase I, phase II and Phase III. [17].

$$\begin{array}{c} eNPV=\sum_{i=1}^{5}\rho_{i}\sum_{t=1}^{T}\frac{DCF_{i,t}}{{\left(1+r_{d}\right)}^{t}}+\rho_{5}\sum_{j=1}^{5}q_{j}\sum_{t=1}^{T}\frac{CCF_{j,t}}{{\left(1+r_{c}\right)}^{t}} \end{array}$$ (2)

In Eq 2, *ρ*_*i*_ describes the conditional probability of stage of development *i* is the final stage for a product at *i* − 1. *T* is when all future cash flows = 0. *DCF*_*i*,*t*_ is the cash flow at *t* given *i* is the end stage. *r*_*d*_ the discount rate and *j* = 1, …, 5 represents an index of quality (An index of 5 was chosen based on the five categories (dog, below average, average, above average and breakthrough) described by Kellogg & Charnes, 2000, which is more than sufficient for this description of a binomial lattice.). The probability of a drug being of a particular quality is *q*_*j*_. *CCF*_*j*,*t*_ represents the expected cash flow upon commercialization (*t*|*j*) and *r*_*c*_ is the discount rate for *CCF*_*j*,*t*_ [17].

When considering either approach, what we observe is that both rely on the determination of some form of risk-adjusted NPV based on the point at which the valuation is sought. Whilst we appreciate this is an oversimplification of a diverse topic, the challenge with this approach is defining and quantifying risk. Most approaches seek to “sum up” risk. The obvious challenge with this approach is that doesn’t allow for the idiosyncrasies, difficulties and market expectations on certain classes of products and treatment areas of varying impact (how do we value an early-stage acne product alongside an early-stage cancer product based upon a novel antibody using the same benefit/cost analysis paradigm without understanding the risks and the impact this can have on clinical success or survivorship?). In practice, these idiosyncrasies are accounted for by considering comparable deals (stage of development, product type, risk, etc.) to understand what the market is willing to bear for a given product in a given category [19]. With the approach of this research we posit a more comprehensive approach to the intangible behavioural asymmetries that impact valuation through the addition of a proxy for market behaviour derived from public sentiment, relevant to a chosen indication or class of drug. This approach would bring a real-time understanding of the market’s appetite for risk at the time the valuation is sought.

## 3 Understanding market behaviour

In recent years, a plethora of research has looked at sentiment analysis as a proxy for market behaviours [21–23]. Many factors have driven this move towards sentiment analysis including the accessibility of rich sources of data such as Reddit, Facebook and Twitter to name just a few. The accessibility and volume of this data allow researchers the opportunity to gauge public agglomeration of opinions on very specific topics in near real-time settings. Whilst the discussions around the application of sentiment analysis are ongoing and evolving, its impact on the study of market behaviour is clear, where topics such as stock market price evaluation and Bitcoin volatility are areas examined [24, 25].

Behaviour-based asymmetries refer to the disparities in how individuals or entities act or react based on varying levels of information, expectations, and sentiment, significantly influencing market behaviours and decision-making processes. These asymmetries, when applied to market behaviors, particularly in the context of RDCs underscore the importance of understanding nuanced investor sentiments and informational discrepancies to refine valuation methodologies and predict market movements with greater accuracy [8, 26].

When considering biotechnology and pharmaceutical RDCs, a meaningful proxy for market behaviour could prove extremely important. Products born out of these transactions can have vast impacts on quality of life and longevity. These effects encourage engagement and buy-in from diverse stakeholder groups who may be affected by these transactions. Whilst previous work has examined the impact of knowledge asymmetries in the RDC context, little work has been done to date to examine to what extent market behaviour impacts RDC valuation methodologies.

### 3.1 Sentiment analysis

The use of either positive or negative sentiment is sensitive to context and serves different purposes. Negative sentiment can be used in well-developed markets where opinions on a service, product or business can be used to understand if market conditions are skewing negatively on a given topic. This can’t be said for new ventures, products or services with little market exposure or visibility. In this context we look to positive sentiment to gain an understanding of broader market conditions, for example, if we don’t have access to enough data for a particular cancer product, we take our examination a level higher to look at the broader industry that produces these types of products as a whole. This is supported where research looking at the airline KLM demonstrated that increased online engagement with customers through the use of social media leads to greater positive engagement in the intended audiences that engage with KLM on these platforms [27]. This is our approach to this research. We look at positive sentiment in relation to the biotechnology industry from a temporal perspective to ascertain investment appetite [28].

In the given context, focusing predominantly on positive sentiment, rather than incorporating negative sentiment, is strategically justified due to several compelling reasons unique to the biotechnology sector and the nature of emerging markets. Firstly, in these nascent stages, negative sentiment, while informative, can disproportionately affect investor confidence and public perception, potentially stifling innovation and funding opportunities crucial for early-stage research and development. Furthermore, in emerging markets or for new products with limited visibility, the availability of reliable negative sentiment data can be scarce or overly skewed by isolated incidents, making it less reliable for comprehensive market analysis. The emphasis on positive sentiment allows researchers and investors to identify and foster areas of growth and innovation, serving as a barometer for market enthusiasm and potential areas for strategic investment [29, 30].

Additionally, focusing on positive sentiment aligns with the objective of understanding broader industry dynamics and investment trends. This perspective is particularly valuable in assessing the industry’s capacity to attract capital, drive growth, and navigate through the complexities of bringing groundbreaking solutions to market. Thus, while not dismissing the importance of negative sentiment in providing a balanced view, the strategic decision to focus on positive sentiment in this context is informed by the specific characteristics of the biotechnology sector, the nature of emerging markets, and the objective to gauge investment appetite and industry momentum. This approach enables a constructive framework for analysing and interpreting market sentiments, which is pivotal for fostering an environment conducive to innovation and investment in high-risk, high-reward sectors like biotechnology [28, 31].

Integration of sentiment analysis in the pharmaceutical and biotechnology industries has revolutionised the understanding of public opinion and consumer behaviour. Recent studies have used advanced deep learning models and demographic analyses to gauge public sentiment toward COVID-19 vaccines, highlighting the importance of addressing vaccine hesitancy and understanding diverse public perceptions [32]. Tools have also been developed that allow companies to respond quickly to consumer concerns and adapt tailored strategies for the pharmaceutical industry [31]. Sentiment analysis has also shown its potential to detect adverse drug reactions, offering a novel approach to pharmacovigilance [33]. These studies underscore sentiment analysis as a strategic tool in the pharmaceutical and biotechnology sectors, helping to monitor in real time the early detection of health concerns and facilitating targeted communication strategies, thus significantly influencing public health outcomes, corporate strategies, and public opinion in the validity of the application of sentiment analysis to complex research.

## 4 Hypothesis

If the heterogony in RDC deals is present, we would expect to observe distinct latent classes separating RDC transactions impacted by public sentiment from those that are not when considering RDC deal data (***H***_**1**_). We use positive sentiment taken from ten years of Twitter micro-blogs relating to biotechnology and the life sciences as a proxy for market behaviour to capture RDCs that look to the market and comparables to benchmarks RDC valuations as put forward in the current literature. These assumptions extend the expectations put forward in the efficient market theories, where if knowledge asymmetries and bias are controlled, then pricing models should fully reflect this information in the price, and practitioners should only look to market behaviour if these elements remain otherwise uncontrolled and not effectively managed or captured [14].

Hypothesis 1 ***H***_**0**_—RDC deal values show no impact from the application of sentiment as a proxy for behavioural-based market asymmetries.
  ***H***_**1**_—We observe that positive sentiment as a proxy market behaviour is observed where at least one latent class is aligned with the application of our proxy.

## 5 Data

### 5.1 RDC deal data

A sample of RDC data was taken from the Current Partnering database [34]. The Current Partnering database captures publicly disclosed deal data for research and development collaborations between biotechnology firms and pharmaceutical companies. Specifically, we were interested in the total deal value and the stages of development at which the deals were entered into. Our sample spanned RDCs from January 1^st^ 2010 through to June 30^th^ 2021. We down-sampled RDC data to weekly intervals, taking the first observation in a given week, to ensure appropriate scale from a temporal perspective for this research, and in line with other research of this nature.

An obvious concern with data of this type is survivourship bias [9]. Our research failed to find any datasets that capture failed RDCs. Understanding the challenges this phenomenon represents, careful consideration was given to model specification and the application of appropriate control variables to ensure an accurate representation of this field in practice.

Survivourship bias in RDCs skews the analysis towards surviving entities, overlooking the failures that could provide critical insights into the challenges and barriers these collaborations face. To mitigate this bias, a broader methodological approach includes a carefully designed study to indirectly infer the impact of non-surviving entities and enhancing the robustness of conclusions. However, this approach has its limits, as the absence of direct data on failed RDCs inherently constrains the depth of analysis possible, emphasising the need for more comprehensive data collection strategies in future research.

### 5.2 Sentiment analysis data

Sentiment analysis drawn from Twitter data is becoming more commonplace in economic research [35, 36]. The use of Twitter data as a proxy for market behaviour is gaining broader academic acceptance as it continues to prove valuable in difficult-to-understand market settings [37]. Although sentiment analysis is gaining a stronger foothold, the debate is still vigorous around the techniques that should be used to produce a meaningful methodology [38].

Twitter data is captured from micro-blogs in the form of “tweets”. When our data were sampled, tweets were 280 characters in length, including text, audio and video. Since completing the research, this character limit has increased to 10,000 characters per tweet (https://www.theverge.com/2023/4/14/23683082/twitter-blue-10000-character-limit-bold-italic-features-substack-newsletter). Twitter users rely on metadata searches to find topics of interest. These metadata tags are referred to as “hashtags” due to the use of the “#” symbol as a preface for any metadata tags attached to the tweet. We used an academic account on Twitter to perform hashtag searches on all tweets of interest going back to the beginning of 2010. The Twitter database extends back to its inception in 2006. Python code was produced to sample n = 10,000 tweets per week through the Twitter API from the 27th of November 2009 through to the 6th of June 2021. The total sample size was n = 5,606,429 tweets for the period described.

Tweetroot was used to produce the primary search terms for the research. Tweetroot is a word cloud production tool that samples like terms from Twitter to refine hashtag searches. Word clouds were seeded with the following terms: “biotech”, “biotechnology”, “Biotech” and “Biotechnology”. After multiple iterations, n = 22 hashtags were captured that were common to each word cloud and the initial search terms.

## 6 Methodology

We seek to understand if behavioural biases can be observed in the analysis of latent classes in RDC deal data. We do this through the application of finite mixture model analysis to total deal values in biotechnology and pharmaceutical RDCs. Mixture models are widely used in a myriad of disciplines, from marine mammal movement, to hospitalisation duration, to the study financial markets, and are considered robust where data is heterogeneous [39, 40]. As the effect we seek to observe is behavioural, we include positive sentiment taken from microblogging on the Twitter platform as a proxy for market sentiment. We include sentiment along with the clinical stage of development (Discovery, Preclinical, Phase I, Phase II and Phase III) to provide a detailed analogue of the features which influence RDC deal-making/doing. Along with this primary model, we also produce several models to explore what impact, if any, the frequency of positive, negative and neutral sentiment tweets per week may have on the models output. In addition to this, we also explore what impact the strength, or magnitude, of the sentiment score may have on the output of the model.

In addressing the challenge of reverse causation in temporal data analysis with finite mixture models, a pivotal strategy is to use each identifiable event only once in the analysis. This approach is specifically effective in scenarios where sentiment or similar factors could influence the lead-up to an event but not its after. By structuring the dataset in this manner, each event is treated as a unique entity, ensuring that any sentiment-driven effects are confined to their temporal context [41].

### 6.1 The model

#### 6.1.1 Specification

As appropriate for this research, we used a conditional model where the conditional probability of an observation in a continuous feature vector is found in *i*th latent class given our **y** vector. We do this through the application of the logistic finite mixture model (**FMM**) described in Eq 3,

$$\begin{array}{c} f(y|\text{x},\text{z},\;\beta ,\theta )=\sum_{k=1}^{K}p_{k}(\text{z},\theta )f_{k}(y|\text{x},\beta_{k}) \end{array}$$ (3)

where *f*(*y*) is the density for deal value, assumed to be Gaussian and conditioned upon the covariates in **x**. With *k* = 1, …, *K* mixing components, each has its own coefficient vector of *β*_*k*_, which is the feature that captures parameter heterogeneity. We employ *K* = 3 mixing components, where the probability of latent class allocation *p*_*k*_(**z**, **θ**) is given by a multinomial logit, with covariates **z** and parameter vector **θ**. This is a simple specification based upon *n* = 3 latent classes that allow for differences in response to our variables of interest. Our model is then estimated through maximum likelihood, with the results in the following section.

$$\begin{array}{c} \varepsilon_{i}=\sum_{k=1}^{K}p_{k}\left({\text{z}}_{\text{i}},\theta \right)\cdot \varepsilon_{i,k} \end{array}$$ (4)

Typically, an FMM captures multiple density functions in a probabilistic setting, where the impacts on the *y* variable come from distinct latent classes (*g*) (In its simplest form, an FMM would employ *K* = 2 mixing components.). In the model we describe, we examine three latent classes, *f*_1_, *f*_2_ and *f*_3_, where the proportions of each density to the total sampled data are described as *π*_1_, *π*_2_ and *π*_3_. *K* = 4 and *K* = 5 component models were also examined, yet both failed to converge, supporting the fit of the *K* = 3 model used (To confirm the validity of the approach and the application of positive sentiment in isolation, a suite of models were examined. These models included each polarity of sentiment in isolation (positive, negative and neutral), pairwise groupings of each sentiment class and finally a combination of all three. These permutations were all tested at *K* = 4 and *K* = 5. All models failed to converge, providing confidence in methodology chosen.). The marginal likelihood is described as follows:

$$\begin{array}{c} L_{c}\left(\theta \right)=\sum_{i=1}^{g}\pi_{i}f_{i}\left(\text{y}|\text{x},c_{i}=1,\theta \right) \end{array}$$ (5)

where **θ** is a vector of the model parameters. The **y** vector captures the observed response variable of RDC deal value and **x** is the vector of independent variables, which include positive sentiment and binary variables for the clinical stage of development. **c**′ is the vector of latent class indicators (*c*_1_, …, *c*_*g*_), which when *c*_*i*_ = 1 all remaining observations of *c* are equal to zero. *π*_*i*_ is the probability of the *i*th latent class and *f*_*i*_(⋅) describes the conditional probability density function in the *i*th class.

The joint density function for a given observation is

$$\begin{array}{c} f_{i}\left(\text{y}|\text{x},\theta \right)=\prod_{j=1}^{n}f_{ij}\left(y_{ij}|\text{x},\theta \right) \end{array}$$ (6)

and we model the dependence of *y*_*ij*_ on **x** through the following linear assumption of

$$\begin{array}{c} z_{ij}={\text{x}}^{\prime }\beta_{ij} \end{array}$$ (7)

where ***β***_*ij*_ is the vector of coefficients for *y*_*ij*_ and thus *f*_*i*_(**y**|**x**, **θ**) can be specified as *f*_*i*_(**y**|**z**_*i*_, θ) and *f*_*ij*_(*y*_*ij*_|**x**, θ) as *f*_*ij*_(*y*_*ij*_, *z*_*ij*_, θ). The likelihood for a given observation is described in Eq 8.

$$\begin{array}{c} L\left(\theta \right)=\sum_{i=1}^{g}\pi_{i}\prod_{j=1}^{n}f_{ij}\left(y_{ij},z_{ij},\theta \right) \end{array}$$ (8)

#### 6.1.2 Probabilities for latent class allocation

Probabilities for latent classes are produced using multinomial logistic distribution where the *i*th class probability is described as

$$\begin{array}{c} \pi_{i}=Pr\left(c_{i}=1|\text{x}\right)=\frac{exp(z_{i})}{\sum_{j=1}^{g}exp\left(z_{j}\right)} \end{array}$$ (9)

or more simply, in our case, if we were to use a full mixture model we would observe

$$\begin{array}{c} \begin{array}{c} \pi_{1}=\frac{1}{1+exp\left(\gamma_{2}\right)+exp\left(\gamma_{3}\right)}\\ \pi_{2}=\frac{exp(\gamma_{2})}{1+exp\left(\gamma_{2}\right)+exp\left(\gamma_{3}\right)}\\ \pi_{3}=\frac{exp(\gamma_{3})}{1+exp\left(\gamma_{2}\right)+exp\left(\gamma_{3}\right)} \end{array} \end{array}$$ (10)

where the probability of an observation being in a given class is described in Eqs 9 & 10. As ***γ***_1_ is treated as the base then ***γ***_1_ = 0. The *i*th class linear prediction is

$$\begin{array}{c} z_{i}={\text{x}}^{\prime }\gamma_{i} \end{array}$$ (11)

and ***γ***_*i*_ is the vector of coefficients, therefore, vector θ is derived from ***γ***_*i*_ as the vector of coefficients for the *i*th latent classes and ***β***_*ij*_ is the vector of coefficients for *y*_*ij*_.

#### 6.1.3 Expectation maximisation

Prior to maximising likelihoods, the starting values are refined using an expectation maximisation (**EM**) algorithm. EM is an iterative method used to estimate parameters in models that are examining unobserved latent classes. The EM algorithm operates in two phases, expectation (**E**) and maximisation (**M**), to compute the expected log-likelihood which is defined as

$$\begin{array}{c} L\left(\theta \right)=\prod_{i=1}^{g}\{\pi_{i}f_{i}\left(\text{y},{\text{z}}_{i},\theta \right){\}}^{c_{i}} \end{array}$$ (12)

where the log-likelihood is expressed as

$$\begin{array}{c} logL\left(\theta \right)=\sum_{i=1}^{g}c_{i}\{log\pi_{i}+log\;f_{i}\left(\text{y},{\text{z}}_{i},\theta \right)\} \end{array}$$ (13)

and to realise the form of the expected log-likelihood we look to the linear function of the expectation (**E**) step as

$$\begin{array}{c} E\left(c_{i}|\text{y},\text{x},\theta_{\left(k\right)}\right)=\frac{\pi_{i}f_{i}\left(\text{y},{\text{z}}_{i},\theta_{\left(k\right)}\right)}{\sum_{j=1}^{g}\pi_{j}f_{j}\left(\text{y},{\text{z}}_{j},\theta_{\left(k\right)}\right)} \end{array}$$ (14)

In this scenario we describe the posterior probability as *p*_*i*_, with the log-likelihood for an observation being

$$\begin{array}{c} Q\left(\theta |\theta_{\left(k\right)}\right)=\sum_{j=1}^{g}p_{i}\{log\pi_{i}+log\;f_{i}\left(\text{y},{\text{z}}_{i},\theta \right)\} \end{array}$$ (15)

where *Q*(θ|θ_(*k*)_) is a function of θ_(*k*)_ through *p*_*i*_, and the maximisation (**M**) step maximises *Q*(θ|θ_(*k*)_) considering θ to find θ_(*k*+1)_.

#### 6.1.4 Regression for latent class predictions

Within latent class predictions are made with a standard linear regression model using

$$\begin{array}{c} \tilde{\mu }=\sum_{i=1}^{g}\tilde{\pi_{i}}\;\hat{\mu_{i}} \end{array}$$ (16)

where $\hat{\mu_{i}}$ is the predicted mean of *y* in the *i*th latent class and $\tilde{\pi_{i}}$ is the predicted posterior probability for the *i*th latent class.

### 6.2 Software

This research used STATA 17, a robust statistical software package, to perform the modelling outlined in this methodology. A Logit Finite Mixture Model was specified to estimate the probability of varying levels of deal valuation based on public sentiment and the development stage of the RDCs.

## 7 Results

As discussed in later in section 6, we considered the impact of positive sentiment derived from biotechnology and pharmaceutical-specific tweets sampled from Twitter’s database along five stages of clinical development (Discovery, Preclinical, Phase I, Phase II and Phase III) to examine if latent classes are present in RDC deal value based upon sentiment used as a proxy for market behaviour. 88% (534 of 602) of the tweets used in this research are significantly positive with an *α* of 5% with a *p*-value of 0.000. This intensity was confirmed with a pairwise *t*-test that compared positive and negative sentiment per week.The terms captured for the search are described in Table 1. The descriptive statistics for these variables can be found in Table 2. As can be seen, Total Deal Value (log) and positive sentiment are continuous variables, with the clinical stages of development dummy, binary variables. Log conversion was applied to the Total Deal Value data to address the data’s right skew. This transformation aims to linearise the data and stabilize the variance.

**Table 1:** Hashtag (#) search terms. Word clouds were produced using Tweetroot using the terms—“biotech”, “biotechnology”, “Biotech” and “Biotechnology”. A total of n = 22 terms were captured that were common to each word cloud and the initial search terms.

#pharma	#therapeutics	#pharmaceutical
#medicaldevices	#healthcare	#therapy
#biotech	#pharmiweb	#clinical
#lifescience	#biotechnology	#pharmaceuticals
#vaccine	#diagnostics	#researchers
#lifesciences	#diagnostic	#medtech
#medicine	#clinicalresearch	#clinicaltrials
#clinicaltrial		

**Table 2:** Descriptive statistics. Descriptive statistics for the features used in the model described in Eq 16. As we see, the dependent variable of Total Deal Value and the independent variable of positive sentiment are continuous, whilst the independent variables representing stage of development are binary.

	Total Deal Value (log)	Positive Sentiment	Discovery	Preclinical	Phase1	Phase2	Phase3
N	444	444	246	68	40	31	14
Mean	4.359	0.098	0.554	0.153	0.090	0.070	0.032
SD	2.392	0.010	0.498	0.361	0.287	0.255	0.175
Min	-2.303	0.057	0	0	0	0	0
Max	8.752	0.125	1	1	1	1	1

**Fig 1:**
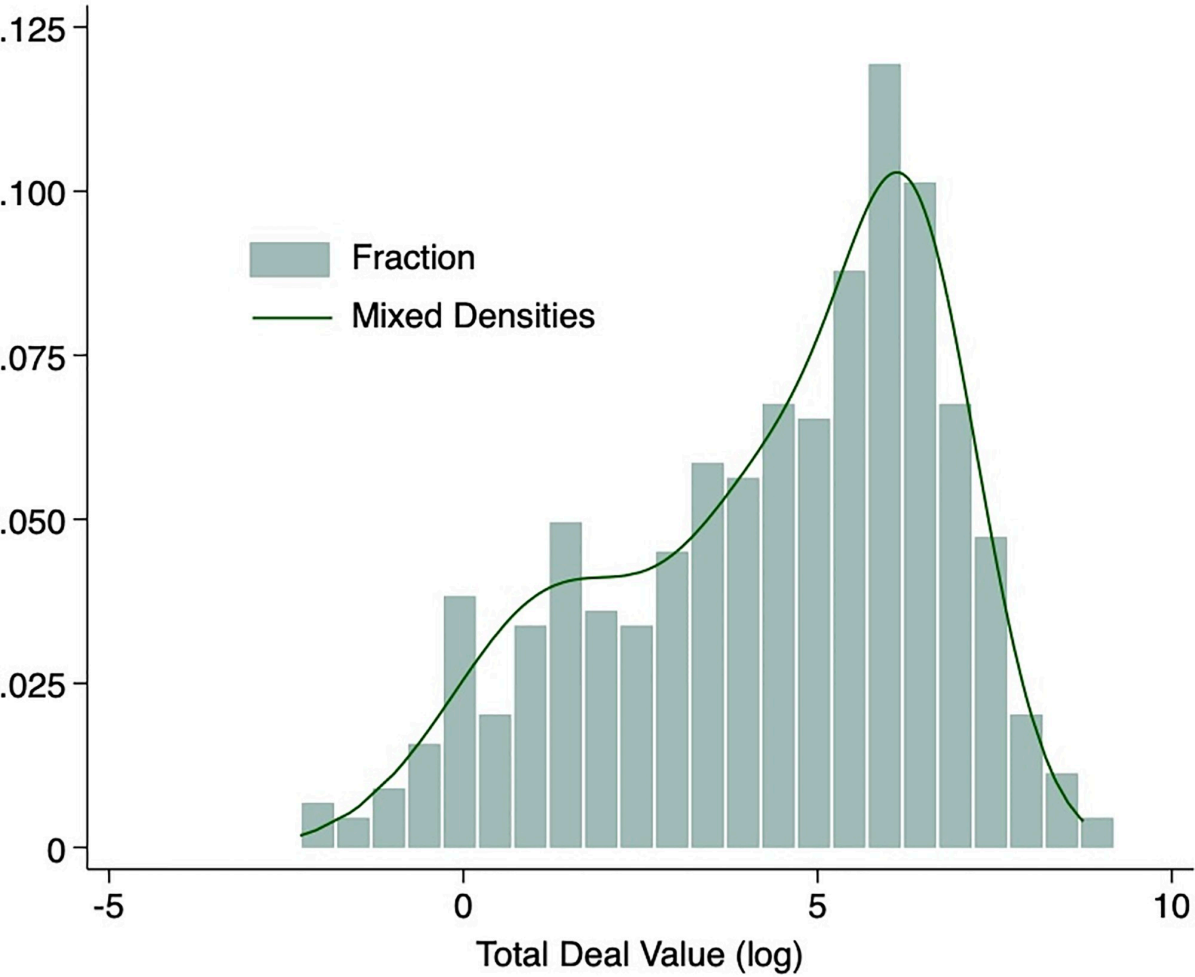
Latent class density. A mixed density of the three latent classes was overlaid on a histogram of the primary Total Deal Value data to visualise if the data displayed a propensity toward being multimodal. As displayed, we see multimodal attributes in the left tails of both the histogram and the mixed densities plots making this data a good candidate for mixture modelling.

A histogram of the primary dependent variable of Total Deal Value and a mixed density plot of the three latent classes were produced and overlaid in Fig 1 for fit. As can be observed, both plots display multimodal attributes as expected. Two peaks can be seen in the left tail of the distribution in the Total Deal Value data, which is reflected in the abnormal left tail of the mixed densities plot. Considering the distribution and the heterogeneous composition of the data, a typical OLS approach would be unsuitable. Both of these plots describe data that would otherwise be considered heterogeneous and non-Gaussian when considered in its entirety, supporting the exploration of the data through the application of a mixture model as described.

Based on the assumptions taken from initial observations of the data, a three-component model was considered most appropriate as discussed in section 6 of this manuscript. Table 3 presents the results of the three-component, finite mixture model. A multinomial logit was employed to predict class membership to ensure the model accounts for unobserved heterogeneity. This was chosen as a logit-based FMM is better suited to modelling class allocation of binary variables such as stage of development used in this research. Total Deal Value is used to specify the class probability for the logit FMM model. Latent classes 2 & 3 are significant when considered against the base outcome at an *α* of 5%. Class 2 & 3 display a downward characteristic in their coefficients with values of -8.6953 and -6.2622 respectively. Clusters were analysed and silhouette scores produced where K2 scored 0.68 and K3 0.80. Both clusters demonstrate solid cohesion and meaningful separation.

**Table 3:** Finite mixture model. Table 3 captures the results of the three component model outlined in the methodology, where a multinomial logit was used to predict class membership based on Total Deal Value. The finite mixture model demonstrates a downward trend in total deal value from class 1. This effect is most prominent in class 2 with a coefficient of -8.6953 on total deal value, -6.2622 for total deal value in class 3.

	Coefficient	Robust std. err.	*z*	*P* > *z*	[95% conf. interval]	
**1.Class**						
**2.Class**						
Total Deal Value (log)	-8.6953	3.563569	-2.44	0.015	-15.67977	-1.710834
Constant	49.4085	23.81555	2.07	0.038	2.730874	96.08613
**3.Class**						
Total Deal Value (log)	-6.262213	2.91269	-2.15	0.032	-11.97098	-0.5534465
Constant	38.34309	19.79684	1.94	0.053	-0.4580124	77.14419

Table 4 displays the outputs of our base outcome, or latent class 1. Latent class 1 finds that positive sentiment is insignificant against Total Deal Value. When we examine the dummies for clinical stage of development, we see that all stages of development are significant at an *α* of 1%. The impact of each of these stages of development appears to be similar in their impact on Total Deal Value. Posterior probability estimations find that latent class 1 exclusively captures RDCs that took place at the Discovery stage.

**Table 4:** Latent class 1. When considering the class allocation table (Table 7), Latent class 1 captures RDCs classed as preclinical. These results demonstrate that these early transactions with long lead times appear to be otherwise unaffected by sentiment due to the lead times for these products to reach the market.

	Coefficient	Robust std. err.	*z*	*P* > *z*	[95% conf. interval]	
Positive Sentiment	-3.735	14.870	-0.250	0.802	-32.879	25.409
Discovery	7.367	1.002	7.350	0.000	5.403	9.331
Preclinical	7.031	0.996	7.060	0.000	5.078	8.984
Phase1	7.177	0.954	7.530	0.000	5.308	9.046
Phase2	7.115	0.912	7.800	0.000	5.327	8.902
Phase3	7.359	0.975	7.540	0.000	5.447	9.270
var(Total Deal Value (log))	0.381	0.159			0.168	0.865

In Table 5 we observe behaviour that is holistically different to that observed in latent class 1. Where latent class 1 captured observations at the Discovery stage of clinical development, latent class 2 is discrete also in its representation of Preclinical RDCs. Positive sentiment is significant at an *α* of 1%, and the magnitude of the effect at 29.865 inspires confidence in the allocation of this class. Preclinical, Phase I and Phase III are significant at an *α* of 5%. Contrary to latent class 1, these three stages of development trend downward against Total Deal Value.

**Table 5:** Latent class 2. Latent class 2 exclusively captures products that were in preclinical development when transacted as captured in the class allocation table (Table 7). These transactions demonstrate the importance of public sentiment on early-stage development.

	Coefficient	Robust std. err.	*z*	*P* > *z*	[95% conf. interval]	
Positive Sentiment	29.865	3.182	9.390	0.000	23.630	36.101
Discovery	-0.784	0.610	-1.280	0.199	-1.980	0.413
Preclinical	-1.097	0.541	-2.030	0.042	-2.157	-0.038
Phase I	-1.094	0.531	-2.060	0.039	-2.135	-0.054
Phase II	-0.583	0.860	-0.680	0.498	-2.269	1.103
Phase III	-2.027	0.668	-3.040	0.002	-3.335	-0.718
var(Total Deal Value (log))	3.0265	0.462			2.243	4.083

When considering Table 6 we observe that latent class 3 caters exclusively to deals in clinical development—Phase I, Phase II & Phase III. Like latent class 1, positive sentiment fails to demonstrate significance for these stages of development, and like latent class 1, all binary independent variables for stage of development are significant at an *α* of 1%. The magnitude of these coefficients whilst meaningful, are less prominent than those observed for the discovery stage of development dummies in latent class 1, ranging from 4.676 to 5.003.

**Table 6:** Latent class 3. Latent class 3 makes up the balance of transactions observed and are exclusive to clinical development (clinical trial phases I, II & III). Unlike latent class 2 however, these transactions also appear to be unaffected by sentiment. Of note, a large portion of these transactions are in phase I, which is also considered early development, and we consider this class capturing transactions that don’t fall into traditional assumptions of “early development”.

	Coefficient	Robust std. err.	*z*	*P* > *z*	[95% conf. interval]	
Positive Sentiment	5.960	10.249	0.580	0.561	-14.127	26.048
Discovery	4.676	1.555	3.010	0.003	1.629	7.722
Preclinical	4.783	1.575	3.040	0.002	1.697	7.870
Phase1	5.003	1.684	2.970	0.003	1.702	8.304
Phase2	4.855	1.795	2.700	0.007	1.337	8.374
Phase3	4.916	1.120	4.390	0.000	2.721	7.111
var(Total Deal Value (log))	0.290	0.091			0.157	0.536

These results find three distinct sub-populations within the data studied. Latent class 1 captures RDCs at the earliest stage of development. Latent class 2 captures RDCs that have survived initial proof of concept and are otherwise ready to progress toward the more complex clinical trials, but still otherwise in the laboratory. Latent class 3 agglomerates all clinical stage (Phase I, Phase II & Phase III) RDCs. In the next section, we discuss what these findings mean and how researchers and practitioners can use this information when considering RDC in future settings—academic and commercial alike. We considered that there may be an impact imparted on the approaches chosen from both the frequency of the tweets per sampling period along with the strength, or magnitude, of the sentiment score. These examinations did not produce meaningful results, which confirms the approach described. The results of these models can be found in Appendix 5.2.

## 8 Discussion

Various research considering RDC in practice has looked at the use of comparables to manage for knowledge asymmetries and bias in RDC market behaviour [42, 43]. Given the diversity of both the products in development and the stage at which the products are transacted, along with the rapid evolution of the industry since the early 1990s, the field requires a more astute means of holistically valuing biotechnology and pharmaceutical RDCs [44]. This research has explored a method to provide a quantifiable measure of sentiment analysis as a proxy for market behaviour which can be used to mitigate the challenges faced by practitioners as they seek to understand market behaviour in the RDC valuation paradigm.

Using a comprehensive and representative dataset, we find that public sentiment surrounding the biotechnology and pharmaceutical industries used as a proxy for market behaviour can be used to classify transactions that are most likely to be impacted by market behaviours. As outlined earlier, This research finds that deals at the Preclinical stage of development are impacted by positive sentiment. Our empirical analysis demonstrates that the data relating to RDCs is multimodal, which supports the use of an FMM to preserve the inherent heterogeneity within the dataset. To this end, a conditional (multinomial logit) model with three components was specified for our research.

One of the most interesting outcomes of this research is the discrete allocation of observations to latent classes based upon stage of development. As observed in Table 7, all Discovery stage observations are captured by latent class 1. Latent class 2 captures all Preclinical stage observations and all remaining deals in the various stages of clinical development are found in latent class 3. Sentiment as a proxy for market behaviour also appears to be discrete in that it appears to impart an effect on Preclinical RDCs exclusively. We posit this is due to the information available to the public and the de-risking of projects as they progress through clinical trials. In the context of biotechnology and pharmaceutical RDCs, a large proportion of Discovery deals have little IP protection and publication on the underlying technology is limited. This generally means that discovery stage RDCs have little to no public disclosures for the market to rely upon [45]. With no disclosure, public sentiment can not be relied upon, and as we observed, there is insignificant impact from positive sentiment at the Discovery stage. In clinical products, RDCs are less impacted by market behaviour and knowledge asymmetries are lessened as disclosures increase and the products and their inherent risks are better understood at a fundamental and commercial level [46, 47]. This allows for more traditional valuation methodologies to hold true in clinical-stage RDCs.

**Table 7:** Class allocation per stage. Class allocation based upon the specified model provide a discrete understanding of how the market reacts to RDCs at varying stages of development. As we observe, discovery RDCs are exclusive to latent class 1, preclinical to latent class 2 and the clinical RDCs to Latent class 3.

	Class 1	Class 2	Class 3
Discovery	246	0	0
Preclinical	0	68	0
Phase I	0	0	40
Phase II	0	0	31
Phase III	0	0	14

With these effects in mind, we find that preclinical deals see a meaningful impact from market behaviour. We believe this is because preclinical products have had enough public disclosure in the form of news and publications to allow the market to form an opinion on the veracity of the claims being made. At the same time, the drugs lack the clinical development needed to offset the knowledge asymmetries as we observe in the clinical products captured in latent class 3. We believe this is why we see such a significant of impact positive sentiment in latent class 2 alone.

## 9 Conclusion

The key points of this research are as follows:

1. **Objective and Methodology**: The study aimed to investigate the use of positive public sentiment as a proxy for market behaviours and to understand what impact this has on RDC valuations.
2. **Model Efficacy**: The model effectively captured the impact of positive sentiment on RDCs and the grouping of knowledge asymmetries based on development stages in each latent class.
3. **Significance of Research**: The findings validate the use of positive public sentiment as a proxy for market behaviour in preclinical RDCs. This is significant as it addresses the challenges of valuing biotechnology and pharmaceutical products, given the complex market behaviours.
4. **Acknowledgment of Limitations**: We recognise the potential influence of survivourship bias and other external factors not included in the study, suggesting a cautious interpretation of the results.

This research sought to examine the use of positive public sentiment as a proxy for market behaviours and knowledge asymmetries present in RDCs in practice. Whilst many methods exist for the valuation of biotechnology and pharmaceutical products, the challenges stemming from the understanding of broader market behaviours persist. Our research is impactful as our findings support our choice of proxy for market behaviour for RDCs at the Preclinical stage of development. The latent groups we observe as part of the outputs of this research support the use of finite mixture analysis when considering the discrete nature of these transactions. Whilst the model demonstrated efficacy in capturing RDCs impacted by positive sentiment as a proxy for market behaviours, it also captures the expected grouping of knowledge asymmetries based upon the stage of development in each Latent class.

Although we cannot rule out the effects of survivorship bias or other exogenous factors not considered by this research, we consider the nature of the outcomes of this research compelling, and a justification for further research in this evolving space. As one would expect, the very challenges facing practitioners valuing RDCs impact researchers examining the phenomenon. Further research would benefit from an understanding of the factors that impact RDCs who don’t find RDC partners and otherwise fail to meet clinical milestones due to a lack of partnership. Understanding these biases could go a long way to shape and inform future research.

Our research has gone beyond the typical understanding of RDC value and provides an understanding of how practitioners and policymakers could benefit from a more holistic understanding of market conditions and their inherent impact on RDC valuations. Future work could look to explore the impact of sentiment on RDCs at a firm level, more specifically, how firm-level disclosures impact sentiment and thus RDC values throughout the product development lifecycle.
